# Extraction of time-related expressions using text mining with application to Hebrew

**DOI:** 10.1371/journal.pone.0293196

**Published:** 2024-02-23

**Authors:** Dror Mughaz, Yaakov HaCohen-Kerner, Dov Gabbay

**Affiliations:** 1 Dept. of Computer Science, Jerusalem College of Technology–Lev Academic Center, Jerusalem, Israel; 2 Dept. of Computer Science, Bar-Ilan University, Ramat-Gan, Israel; 3 Dep. of Informatics, Kings College London, Strand, London, United Kingdom; Industrial University of Ho Chi Minh City, VIET NAM

## Abstract

In this research, we extract time-related expressions from a rabbinic text in a semi-automatic manner. These expressions usually appear next to rabbinic references (name / nickname / acronym / book-name). The first step toward our goal is to find all the expressions near references in the corpus. However, not all of the phrases around the references are time-related expressions. Therefore, these phrases are initially considered to be potential time-related expressions. To extract the time-related expressions, we formulate two new statistical functions, and we use screening and heuristic methods. We tested these statistical functions, grammatical screenings, and heuristic methods on a corpus containing responsa documents. In this corpus, many rabbinic citations are known and marked. The statistical functions and the screening methods filtered the potential time-related expressions and reduced 99.88% of the initial expressions (from 484,681 to 575).

## Introduction

Keywords and keyphrases are some of the most important tools in information technologies e.g., information extraction (IE) and information retrieval (IR). The information that keywords and keyphrases possess is substantial; they provide the “building-blocks” in searching and mining many different fields, including the industrial, legal, and academic fields [1–6]. Therefore, the rapid growth of automatic extraction and analysis of keywords and keyphrases is inevitable. Machine learning, electronic corpora, semantic relations and classification, plagiarism detection, and web search engines [7–11] all utilize keywords and keyphrases as part of their functionality. For example, clustering keywords and keyphrases provide hints or insight into the semantics of expressions, sentences, or paragraphs [12].

The need for keywords and keyphrases is important not only for industrial or scientific papers but also for Hebrew rabbinical responsa (rabbinic scholars who wrote answers in response to Jewish legal questions) [4]. Semitic languages are quite different from Latin languages; therefore, Hebrew and especially responsa documents, or Semitic language, will have dissimilar processing from the English language. One difference is that documents written in Latin languages are written from left-to-right, while those written in Hebrew are written from right-to-left [13]. Another difference is that Hebrew and Aramaic languages can appear together in a text, which adds to the complex morphology of Semitic languages (e.g., keywords and keyphrases can exist with several forms of prefixes "and when in …", "and when …", "and …", "when …", "in …"). One of the effects of this complex morphology is ambiguous keywords and keyphrases in Hebrew and Aramaic texts [14]. The third difference is that in Rabbinic documents, which are written in Hebrew and Aramaic, there is much more frequent use of abbreviations and acronyms than in regular Hebrew documents [15]. A study conducted by [16] demonstrated that the manual disambiguation of an acronym is an extremely time-consuming process, and it is a very difficult task even for a professional. For these reasons, keywords and keyphrases that appear in rabbinic responsa documents are much more difficult to extract than keywords and keyphrases in academic papers or any other English article.

In this study, we rank and extract a set of keywords and keyphrases from rabbinical texts written in Hebrew and Aramaic. These texts have been written by 24 different authors over the past 231 years (see S1 Appendix). The set of expressions we are looking for is unique because it contains a meaning of time (an explanation will be presented below); we call these expressions time-related expressions (TREs). Some will treat these expressions as expressions of respect for one another, and some will interpret these expressions as expressions that contain the meaning of time. For example, let us consider זצוק"ל, which means “may the righteous and holy be of blessed memory”. On the one hand, the author refers to another person as righteous and holy, and at the same time says that the mentioned person has already passed away. These TREs can help us build a timeline, determine when certain books were written, determine whether texts were edited or forged, and even help us to associate an anonymous book with an author.

The retrieval of TREs is accomplished in two steps. The first step involves extracting potential time-related expressions (PTREs) in an unsupervised manner, and then rating and filtering them. Extracting, rating, and filtering will be conducted using new statistical formulas (section 4.3) that we have developed for this research, and the screening methods (section 5.1) will only filter the PTREs. Text is a one-dimensional environment, and because the general idea of one of the statistical formulas (for extracting and ranking TREs) is that the TREs appear next to references/citations of other names or nicknames, it can be applied to more than one dimension. A simple adaptation of the formula for 2D can involve extracting and rating a set of pixels within an image that appears around a known pattern.

Time expressions are characteristics that can help us to place different parts of text over a time axis. These expirations can be used to sort ancient books/responses/writings in chronological order and, in some cases, help to identify anonymous authors.

The motivation of this study is to extract time-related characteristics to help automate the dating of old texts in general, specifically rabbinic texts. For example, if in the text "Z", the name of Mr. Y appears with the expression "of blessed memory", we know that this text was written after the death of Mr. Y. In addition, if this text contains the phrase, "my friend Mr. X, may G-D preserve him and grant him life” we know that this text was written while Mr. X was still alive. Assuming that we have the birth and death years of Mr. X and Mr. Y, we can estimate at what interval of time the "Z" text was written [3, 4]. Of course, a text can contain references to many more people, which can help to more precisely evaluate the time interval.

The contributions of this study are described as follows. In this study, we intelligently search for keyphrases (the length of words of each expression can be different) in a small text window (if we use a wide window, it would encompass numerous phrases positioned "close" to mentions that do not pertain to our specific target expressions). While most of the studies in this field look for keywords/phrases throughout the entire document, we are looking for words only next to references. Most studies look for documents with a common subject by extracting common words within the documents. Also, our task is unique, it does not deal with explicit time expressions but with the extraction of phrases that they special honorific expressions. These expressions contain a latent meaning of time. With the help of these expressions, we can deduce a relative time estimate (i.g., if it is written in the text of “X”, that the person named "Z" died, we can know that this text was written after "Z" passed away).

For this purpose, we have developed two statistical formulas to filter and rank candidates of time-related expressions. As far as we know, we are the first to extract time-related expressions (that implies Time) in an unsupervised manner from Semitic languages in general and Hebrew/Aramaic in particular. Relatively few studies have been conducted on these languages, and this is another obstacle/difficulty we need to overcome. We show how to make unique use of grammatical affixes in an environment with complex language morphology. Many of the affixes encapsulate stop words.

This manuscript is organized as follows. The **Related works** section provides background concerning the extraction and analysis of keyphrases. The **Examples of Responsa texts with citations and TREs** section introduces examples of rabbinic texts. **The extraction and rank of PTREs** presents the extraction and rank of PTREs. **Experimental Results** presents experimental results and analysis, and in **Summary, Conclusions, and Future Work** summarizes our major findings and suggests ideas for future work.

## Related work

Extracting keyphrases is a necessary step in several Natural Language Processing (NLP) tasks, such as clustering, ontology, and summary [8, 17]. In other NLP tasks, there is a real need for big data. Therefore, when providing keywords and keyphrases for these tasks, they become much easier and faster [18].

There are many approaches to extracting keywords and keyphrases that exist in the professional literature. These approaches can be divided into two categories: supervised and unsupervised. These approaches were applied to different types of domains, including news texts, scientific articles, abstracts, webpages, and meeting transcripts [19–24].

Supervised learning techniques generate prediction models by learning from a large number of training samples, each labeled with a ground-truth result, especially in tasks like classification and regression [25]. In supervised learning, an algorithm can generalize knowledge from existing examples. This ability allows anticipating new unforeseen circumstances [26]. However, the labeling process usually requires a great deal of effort, and sometimes human labeling is required [25, 27]. Manual labeling requires time and financial resources [25, 27]. The majority of the effective approaches, such as deep learning, need the provision of ground-truth labels for a large data set. However, in many tasks, obtaining good tagging information might be challenging. As a result, ML approaches should be mixed with unsupervised learning techniques [28]. Unsupervised learning requires only the data with no need to pre-label samples [29].

There are unsupervised approaches for extracting keywords, these approaches have been gaining momentum in recent years. In contrast to supervised approaches, these approaches, do not require human or expert supervision to check the results, which is an expensive and sometimes impractical endeavor. The unsupervised approach to extracting expressions is defined as a grading problem, each word receives a score based on functions such as TFIDF [30] or PageRank [22]. The score of an expression is usually calculated by adding the score of the words that make up the expression [22, 31], and the expressions that received the highest scores are defined as the keyphrases of the document. One of the problems with this keyphrase method is the length of the expression. Because the score of the expression is a sum of the scores of each word, longer terms will receive a higher ranking (the expression “w1 w2” will get a lower score than the expression “w1 w2 w3”).

The unsupervised methods mentioned above are efficient and competitive with the supervised methods [32, 33], and therefore have become more usable today. The PageRank algorithm [22] is a very useful tool for extracting keyphrases in advanced models. Other methods convert a document to a graph, and the most central methods represent the most important words. These methods build directed graphs for the document so that each word is a vertex in the graph and then measures the word centrality by betweenness or closeness [34, 35]. They are also used to extract keyphrases. Campos et al. (2020), [36], presented an unsupervised keyword extraction model (called YAKE!), which is based on statistical text features that are extracted from single documents. Their model does not need to be trained on any set of documents, nor does it depend on dictionaries, domains, external corpora, language, or text size. To demonstrate the merits and significance of YAKE!, they compare it against ten state-of-the-art unsupervised approaches and one supervised method. Experimental results against ten state-of-the-art unsupervised approaches that were carried out on more than 20 datasets showed that YAKE! significantly outperforms all other unsupervised methods on texts of different sizes, domains, and languages.

Danesh et al. (2015), [37], initially calculated the weight of each expression using a modified version of the FTIDF, similar to what was done in KP-Miner. The weight calculation was performed using a combination of scores obtained from the modified TFIDF and the position of the first occurrence of the expression in the text. The phrases and their initial weight were incorporated into an algorithm that builds a graph from the words in the document and finally gives a final rating to the keyphrases. They compared their results to KP-Miner and TextRank and they obtained better results from them.

Hulth (2003), [19], extracted keyphrases using a Part-Of-Speech (POS) tagger, to create a set of candidates. Her use of POS was not in a single POS, but in potential POS patterns to find phrases in the text with the potential to be candidates for keyphrases. Using this method of integrating POS as a feature for keyphrase extraction showed a significant improvement in the outcome of these phrases.

Song et al. (2003), [38], developed a method called KPSpotter. This method combines information gain with NLP techniques, such as the first instance of an expression/term and POS tags. In KPSpotter, they have also integrated WordNet, and it improves system accuracy.

Sahrawat et al. (2020), [39], proposed various sequence labeling models using embeddings for keyphrase extraction from scholarly articles. The best results were obtained using a BiLSTM-CRF architecture using domain-specific contextualized embeddings (SciBERT) on three widely used public datasets: Inspec, SemEval, and SemEval-2017. Sun et al. (2020), [40], proposed an unsupervised keyphrase extraction model based on a pre-trained language model called SIFRank, which combines a sentence embedding model called SIF and an autoregressive pre-trained language model ELMo [41]. For long documents, they upgraded SIFRank to SIFRank+ by position-biased weight. Compared to other baseline models, their model achieved state-of-the-art results on three widely used public datasets: Inspec, DUC2001, and SemEval2017. Awan and Beg (2021), [42], proposed TOP-Rank, a model for keyphrase extraction and keyphrase classification. Their extraction model is based on the position of keyphrases in the document and the topical ranking of the keyphrases. Their model ranked the keyphrases using intra-cluster and inter-cluster ranking to identify the top most keyphrases.

Scientific articles contain many references and they create “citation networks”. Caragea et al. (2014), [34], used KEA and Hulth’s methods [19], which are supervised techniques, to seek keyphrases from scientific articles by exploiting the fact that they contain many references. The fact is that citation networks are a type of tool that streams information between scientific articles. Each vertex, i.e., any reference in this type of text, also contains a summary of the article to which the reference was made. Caragea et al. (2014), [34], built a Citation-Enhanced Keyphrase Extraction (CeKE) system that takes advantage of the information contained in references (where there is a summary of the article), as well as information contained in the article itself. First, they extracted candidates to be keyphrases by the following process: (1) the POS filter was used to retrieve nouns and adjectives, (2) a stemmer was applied to each word, (3) adjacent words were chained to an n-gram, and (4) phrases that end in an adjective and words that are adjectives were deleted. Subsequently, they ran their database on the World Wide Web (WWW) and knowledge discovery and data mining (KDD) datasets and only retrieved keyphrases that were above a very high threshold. They reported better performance than other new supervised and unsupervised methods.

Rabby et al. (2020), [43], proposed an unsupervised keyphrase extraction method called the Tree-based Keyphrase Extraction Technique (TeKET). Their method uses limited statistical knowledge and it is domain and language independent and requires no train data. Experimental results that were carried out on three datasets: SemEval-2010, Theses100, and a German Research Article dataset showed improved performance of the proposed method over other unsupervised methods in terms of precision, recall, and F1 scores.

The existing approaches that we mentioned above look for keyphrases or keywords that relate to the subject of the document to classify the document by subject rather than classify the document by date. To classify a document by topic, use the bag-of-words approach using words that appear throughout the document without reference to their location [44, 45]. To find TRE we need to look for words and phrases in the vicinity of references by authors. We are interested in words/phrases "stored" in the content meaning of time and these keyphrases or keywords are near the names of writers (not every writer’s name has a time word) so we do not go through all the keyphrases or keywords in the documents but only keyphrases or keywords next to writers’ names.

Some articles extract time expressions; however, this research extracts honorific expressions containing hidden time indications without explicit time expressions, such as "of blessed memory" or "rest in peace". Identifying and labeling various temporal elements to enrich the understanding of documents can facilitate tasks like event extraction and summarization. Existing articles from the literature search use explicit time expressions as tags for extraction or classification. These expressions include dates (e.g., "July 15, 2023", "15/07/2023", "2023-07-15"), hours (e.g., "2:30 PM", "14:30"), relative time expressions (e.g., "last Friday", "next week"), days of the week, months, seasons, and other indicators of explicit time.

Lee et al. (2014), [46], presented a new approach that learns to identify and interpret time expressions in natural language. The approach uses a Combinatory Categorial Grammar to construct compositional meaning representations for time expressions. It also uses data, such as the document creation time and the tense of the governing verb, to compute the final time values. Their experiments outperform previous SOTA results, with 13% to 21% error reductions in end-to-end performance. Few articles propose a new method for extracting temporal expressions from text in multiple languages. One of them [47] uses adversarial training to align the embedding spaces of multilingual models, which allows them to share information across languages. This results in a more accurate and robust temporal expression extractor that can be used in various languages. Another study [48] proposes a new framework for temporal expression extraction in low-resource languages. Their framework, called XLTime, uses cross-lingual knowledge transfer to improve the performance of temporal expression extractors in low-resource languages. XLTime achieves this by leveraging a pre-trained multilingual language model to learn shared representations of temporal expressions across languages.

Ma et al. (2022), [49], propose a new method for extracting temporal information from social media messages using the BERT model. The method first uses BERT to encode the social media message and then uses a conditional random field (CRF) to label the temporal expressions in the message. The authors evaluated their method on a dataset of social media messages and achieved SOTA results. Wang et al. (2023), [50], also used BERT. The authors introduce a novel language representation model called BiTimeBERT. BiTimeBERT is trained on a temporal collection of news articles and harnesses distinct temporal signs to construct time-aware language representations:

1. Absolute time expressions (e.g., "February 23, 2007)
2. Relative time expressions in a news article (e.g., "yesterday", "last month")
3. Event time estimation (e.g., "American Revolution", "9/11 attacks")

The experimental results show that BiTimeBERT consistently outperforms BERT, BERT-NYT, and other existing pre-trained models on different downstream NLP tasks and applications for which time is important. BiTimeBERT manages to achieve better results than other models with significant improvements.

A new method for temporal reasoning in natural language text was proposed by Cai et al. (2023), [51]. The method, called Logic Induction Enhanced Contextualized Temporal Reasoning (LECTER), uses (1) temporal dependency inducer, (2) temporal concept defuzzifer, and (3) logic validator. LECTEr can reason temporal concepts, such as "before" and "after", and explain its reasoning process.

In 2023, Miller et al. (2023), [52], proposed a novel end-to-end system for extracting temporal information from clinical text. The system uses a pre-trained transformer encoder to learn contextualized representations of the text and then uses a multi-head attention mechanism to identify the temporal relationships between events and time expressions. The system was evaluated on the THYME corpus, a benchmark dataset for clinical temporal information extraction. The system achieved an F1 score of 0.53 on the in-domain setting and 0.36 on the cross-domain setting. These results outperform the SOTA on this dataset. The proposed system can be used to improve the understanding of clinical data and to support clinical decision-making.

Zhong and Cambria (2023), [53], conducted a comprehensive review of the progress in Time Expression Recognition and Normalization (TERN), primarily focusing on three key approaches: rule-based, machine-learning, and deep learning. The authors also delve into the challenges faced by TERN and explore potential directions for future research. A major obstacle in TERN is the significant variability exhibited by time expressions. These expressions can manifest in diverse forms, encompassing natural language, abbreviations, and acronyms. Furthermore, the meaning of a time expression can shift based on the specific context in which it is used. Machine learning methods have demonstrated their effectiveness in TERN, yet they necessitate large amounts of data. On the other hand, deep learning methods have also proven potent, but they come with the drawbacks of being both data-intensive and computationally expensive.

HaCohen-Kerner et al. (2011), [54], worked on Hebrew-Aramaic rabbinic documents and applied six machine-learning methods for the automatic identification of citations. To accomplish this, they used four types of feature sets, orthographic, quantitative, stop word-based, and n-gram, and then they tested them separately and together. The most successful results were based on a combination of the four feature sets. Their research identified if a sentence included a citation; however, they were not able to recognize the citation itself, nor its position in the sentence.

Mandala et al. (1999), [55], developed a thesaurus that uses the dice coefficient and the relations between words that appear in WordNet. The dice coefficient uses the co-occurrence of words and measures the degree of statistical correlation between them. They found that joint occurrences between words can automatically provide semantic relationships that naturally exist in a human thesaurus. In addition to the success of the co-occurrence method, they reported that the integration with the WordNet information improves the results.

Moghaz et al. (2019), [4], and Mughaz et al. (2014), [56], built a system that evaluates the period in which a writer lived. They built a set of rules/functions that use TREs to find the upper and lower bounds of their years of activity. They examined the rules of rabbinical texts written in the Hebrew-Aramaic language. The TREs are extracted by a "Ping-Pong" boosting algorithm. They took advantage of the fact that such expressions usually appear near citations, and they manually created a very limited list of four different authors and extracted sentences containing the names of those writers. From these sentences, they manually extracted TREs. Next, they searched for sentences containing the TREs they had found in the previous stage. The new sentences contain the names of the writers. From these sentences, they scrapped all the sentences containing the names of authors they had used on previous occasions and pulled out new names. Now, they have returned to searching for sentences containing new authors’ names (the previous step). The algorithm does work, but the obvious disadvantage of the algorithm is the need for manual work and manual testing of all new sentences at each stage.

Most previous works look for expressions/words that characterize a single or cluster of documents containing common information. In this work, we search for keyphrases or keywords that appear close to citations in pre-modern Hebrew-Aramaic texts, and extract TREs from this set. One of the essential differences between citations in these texts and modern texts, especially scientific ones, is that there are no special patterns representing references [57], and no bibliography.

In the context of time expressions, we did not come across work dealing with extracting expressions implying time. All the works dealing with the extraction of time expressions extract explicit time expressions (such as date [in various styles], hour, month, season, Sunday, January, future, past.) It can be said that the expressions we extract provide indirect information that implies time (a complete list appears in S2 Appendix).

Time expressions can be categorized into the following groups:

- An expression of respect implying that the rabbi mentioned had already passed away at the time of writing the text (for example: ז"ל—acronym: of blessed memory, זכרו לברכה—of blessed memory)
- An expression of respect implying that the rabbi mentioned is still alive at the time of writing the text (for example נר"ו—acronym: may G-D preserve him and grant him life, ידידו מוקירו—his dear friend).
- An honorific expression that gives a hint of relative time; that is, it is not possible to know whether the person mentioned is still alive or deceased, but it is possible to know that the writer is older or younger (for example אבי—my father, תלמידי—my students) than the rabbi he mentions in the text.

## Examples of Responsa texts with citations and TREs

Here we present a few snapshots from responsa texts with translations to English to show rabbi names near TREs. The rabbi names are in italics and the TREs are in bold. For example, in the first sample, the rabbi’s name is *Rama"ch* and the TREs are ***move"r*** (before the rabbi’s name) and ***hai"v*** (after the rabbi’s name). We can see that the TREs are tied to the rabbi’s names.

שו"ת שמחת כהן חלק יורה דעה א סימן פו .. וכן הסכים **מו"ר** *הרמ"ך* **הי"ו** להתיר…

### Responsa Simchat Cohen Part Yoreh Dea`a 1 chapter 86

… and agreed **move"r** (acronym: My Teacher and my Rabbi) the *Rama"ch* (acronym: Calfon Moshe HaCohen) **hai"v** (acronym: may G-D preserve him and grant him life) to allow …

שו"ת רבי אליהו מזרחי (הרא"ם) סימן עה

אבל *הרמב"ם* **זכרו לברכה** כתב וכן אם יצאו עשרה בני אדם כאחד ממקו’ למקום וכו’…

### Rabbi Eliyahu Mizrachi (the Ra’am) chapter 75

But the *ramba"m* (acronym: Rabbi Moshe ben Maimon) **zichro livracha** (of blessed memory) wrote as well if ten people left together from place to place etc`…

## Extraction and rank of PTREs

Potential time-related expressions (*PTREs*) are all the terms found near references that might encapsulate some time-meaning in them (explanation and example appeared in section 1). Those expressions might have nothing to do with time, but they still, provide answers for our specific demand.

To gain the ability to extract the PTREs, we marked the references in our text. We used a list of references of known writers, books, or nicknames. This list appears in [57] Mughaz et al. (2017). The marking was done by iterating through the text and marking any known writer, book, or nickname.

To make the PTREs extraction even easier, we divided the text into two parts: sentences with marked references and sentences without marked references (the sentences without marked references may have some references that we failed to mark).

Now, we extract from both sides of the marked references a sequence of words from a defined window size. String extraction was conducted iteratively; with every iteration, the window-size decreased (details will appear below). Each extraction is now PTRE, which potentially has the information we are looking for.

After extracting all the PTREs, we must filter and rank them. The core of the filtering and ranking idea is inspired by the fact that, if a string happened to frequently appears near reference and appears much less frequently in the rest of the corpus, it may be a phrase that we are looking for; however, if the string appears more frequently in areas away from the references, it may be not a phrase that we are searching for. It has a comparable idea, to the dice coefficient [55, 58, 59] but differs from it. The dice coefficient works on the probabilities of pairs of words/samples) that appear on a document and seeks "similarity" between documents based on these words. Our function works on a defined window next to a defined expression, i.e., a citation. Our function is not looking for a relation between different expressions, but to what extent the phrase or word is related to any citation.

### The general algorithm

The general algorithm contains nine stages, which are defined as follows:

1. Extract AN (AN = author names/nicknames/acronyms/book-names) of rabbis/authors.
2. Search for sentences containing AN.
3. Extract strings on the right and left of the ANs of m words (and less, until one word) from these sentences, and enter the extracted strings into a vector.
4. Delete any string within a vector that contains stop words.
5. For each string in the vector, count the occurrences of this string (in the same vector) so that at the end, we will have the number of occurrences of each string in the vector.
6. Delete any strings from the vector whose number of occurrences is less than three.
7. Search in the entire corpus for all the strings that are contained in the vector and count their occurrences.
8. Compute the weight/rank for each string (see below sections 4.3.1 and 4.3.2) in the vector based on the number of occurrences near references vs. not near references.
9. Sort the vector in decreasing order by weight.

**Fig 1:**
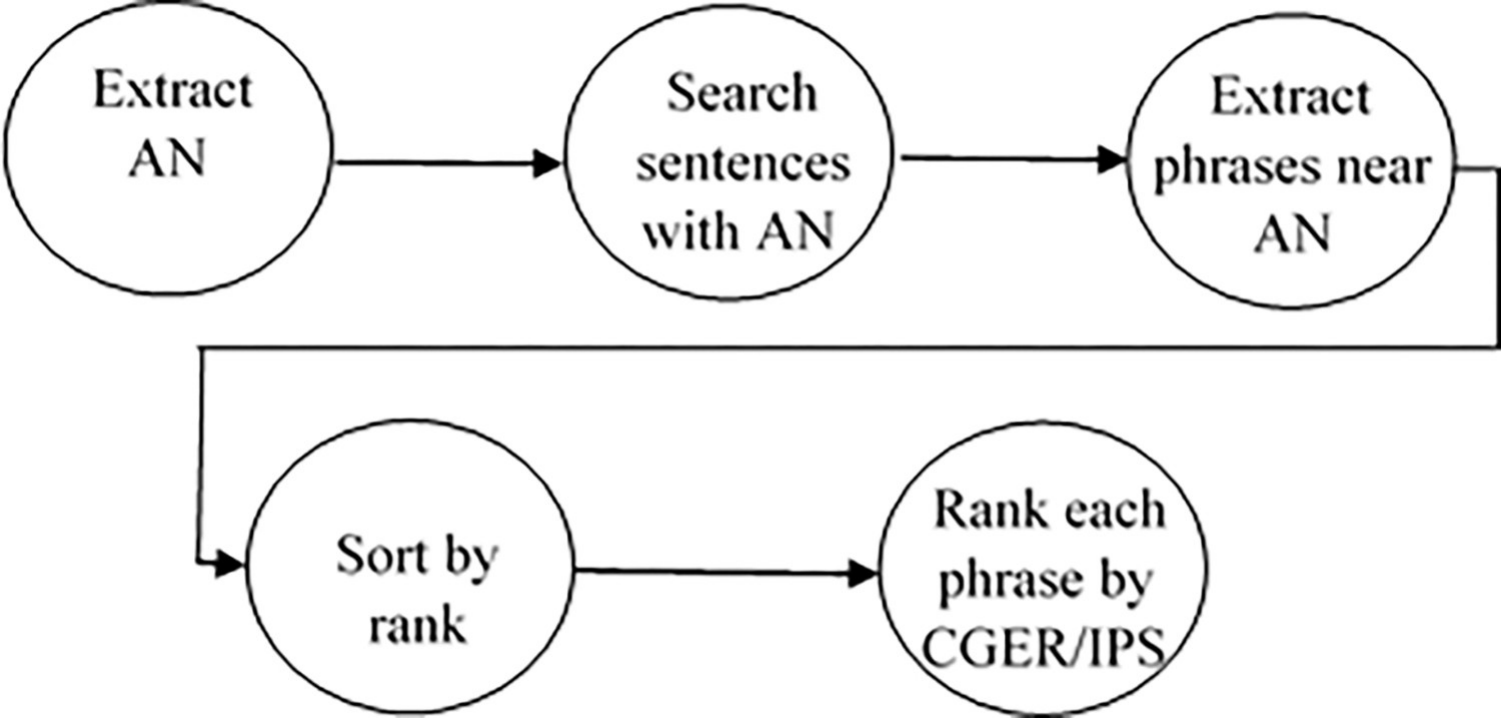
The main steps of the general algorithm.

Diagram of The main steps of the general algorithm appear in Fig 1.

In the next sub-section (4.2), we explain stages 1–7 of the general algorithm in detail. We start with notations and then we write a pseudo code for those stages. In subsection (4.3), we explain stages 8 and 9 in detail. Because stages 8 and 9 contain the statistical functions, which are very important, we include examples with our explanation.

### This sub-section a detailed explanation of stages 1–7 of the general algorithm

Notations:

- DS–the data sets (set of sentences).
- AN–set of phrases of authors’/Names/nicknames/acronyms/abbreviations/book-names.
- SW–set of stop words (including: chapter, section, halacha, Talmud, Gemara, etc.).
- SSV–vector of sub-strings and their occurrences near references and in the whole corpus, i.e., (sub-string, ref-num, corp-num).
- ES–set of extracted sentences that contain the author’s name.

The first step of the algorithm is to find the strings that may be TREs.

Stage (1)

 We took the authors’ names from Mughaz et al. (2017), [55].

Stage (2)

/*Extract from our data-sets (DS) all the sentences, which include the author name (AN) */.

 **for all** *s_i_* ∈ *DS* **do** *⊳* The sentence contains a phrase of an author name

  **if** (∃*n* ∈ *s_i_*: *n* ∈ *AN*) **then**

   *ES ← ES* ∪ {*s_i_*}

  **end if**

**end for**

Stage (3-5)

/*Extract from the extracted sentences (ES) all possible sub-strings of length (i.e., words) to 1 (i.e., word). */

Let SSV be a vector structured of three fields *(str*, *int*, *int)*; the first is a string, i.e., *SSV*.*str*, the second is an integer, i.e., *SSV*.*near*_*ref*_*count* and the third is also an integer, i.e., *SSV*.*corpus*_*count*, (e.g., (abcdef, 5, 7) means that string "abcdef" have 5 instances in the ES and 7 in all of the corpus, i.e., in DS).

**for all** *k_j_* ∈ *ES* **do**

 **for** *len ← m*,1 **do**

 Search in *k_j_* for the *index* of *reference* ∈ *AN str_a_ ← len* words after *reference* index in *k_j_ str_b_ ← len* words before *reference* index in *k_j_*

    *⊳* if *str_a_* contains a stop word or the author’s name

 **if** ∃*w* ∈ {*AN* ∪ *SW*}: *w* = *word* ∈ *str_a_* **then**

  Ignore *str_a_* **else**

  **if** *str_a_* = *SSV i*.*str* **then**     *⊳ str_a_* is string in SSV

  *SSV_i_.near*_*ref*_*count ← SSV_i_.near*_*ref*_*count* + 1 **else**

  *SSV_e_.str ← str_a_       ⊳ SSV_e_* is the end of SSV

  *SSV_e_.near*_*ref*_*count ← SSV_e_.near*_*ref*_*count* + 1

  **end if**

 **end if**

 **Repeat** for *str_b_* **end for end for**

 Stage (6)

/*Delete all strings from SSV with several occurrences less than three */

**for** *i* ← 0,|*SSV* | **do**

 **if** *SSV_i_.near*_*ref*_*count <* 3 **then**

  *SSV ← SSV* \ *SSV_i_* ***⊳*** delete *SSV_i_* from the vector *SSV*

 **end if**

**end for**

 Stage (7)

/*For every *SSV*.*str*, count its occurrences in the entire corpus, i.e., DS */

**for all** *s_i_* ∈ *DS* **do**

 **if** (∃*n* ∈ *s_i_*: *n* ∈ *SSV*.*str*) **then** *⊳* The sentence contains PTREs

  **for** ∀ *SSV_i_.str* = *n* **do**

   *SSV_i_.corpus*_*count ← SSV_i_.corpus*_*count* + 1

  **end for**

 **end if**

**end for**

**Stages (8) and (9)** of the algorithm will be explained in detail in the next section.

### The ranking algorithms

To investigate the statistics of the extracted PTREs, we need to know for each PTRE SSVi.str, the number of times it appears in a sentence near references, i.e., SSVi.near_ref_count, and the number of times it appears in non-nearby references, i.e., SSVi.corpus_count.

A PTRE that many of its appearances are not nearby references and a few of its appearances are nearby references is less likely to be discovered as an actual TRE, i.e., SSVi.near_ref_count <<SSVi.corpus_count.

### The common ground expression ranking formula

The **C**ommon **G**round **E**xpression **R**anking (*CGER*) is a ranking formula that we defined and formulated for this research. It determines the weight of a term by using the number of times it appears near references and the number of times it appears in the **entire corpus** (also not next to references).

The motivation is to classify/rank the PTREs according to the logical assumption that PTREs appear more frequently near references than in the rest of the corpus.

The score range is between zero and one, where zero indicates that there is no correlation and one indicates complete correlation. (In the dilate explanation of stages 3–5 (4.2) we elaborate on the structure of the vector. The *SSV* is a vector structured of three fields *(str*, *int*, *int)*).

Let *x* be *SSV_i_.near_ref_count*

Let *y* be *SSV_i_*.*corpus_count*

Let e be *SSV_i_.str*

Above, at the beginning of section 4 and later, in session 5.3, we mention that there are unmarked references and time-related expressions that were omitted due to the partial marking of references. Because TREs appear near references, we marked all the references that we took from Mughaz et al. (2017), [57]; However, they did not cover all the AN (Authors’/Names/nicknames/ acronyms/abbreviations/book-names). This leads to a scenario where there are unmarked references that have TREs next to them. We count in *SSV_i_.near_ref_count*, i.e., *x*, only expressions that are next to **marked** references, and this led to damage to the value of *x* (explanation is below). This is because TREs that are next to unmarked references count as *SSV_i_*.*corpus_count*, i.e., *y*, and not as *x* where they should count. For example, if TRE ’Z’ appears next to marked references 10 times, x = 10. If TRE ’Z’ also appears next to unmarked references 10 times and an additional 2 more times in the rest of the corpus, y = 10 + 10 + 2. If the unmarked references were marked, as they should be, the statistic was x = 20 and y = 22 instead of what we have now: x = 10 and y = 22. In light of that, we gave a higher weight to *x*. From a sample test we performed, we have seen that the number of these errors is close to the number of *x* (as in the example above), so in the formula, we multiplied the weight of *x* by 2 (notice: *x* is contained in *y*).

The definition of CGER that we defined and formulated is as follows:

$${CGER}_{e}(x,y)=\frac{2*x}{x+y}$$ (1)

For instance:

The excretion *e = “blessed memory”* appears near references 170 times and in the entire corpus (include near references) only 250 times.


$${CGER}_{e}(170,250)=\frac{2*170}{170+250}=0.81$$

We run the CGER function over all *SSV*.*str* to rank all PTRE strings.

CGER has a disadvantage with very low-frequency phrases. In any text, some expressions will appear a small number of times. For example, assume that an expression appeared two times next to references and once in the rest of the corpus; in such a case it will receive a relatively high rating. A much more serious problem is when there is a typo. Typo errors exist, but if such a mistake appears next to the references, it is reasonable to assume that in the rest of the corpus, it will not appear at all and then will receive the maximum rating. To minimize such problems, we ignored expressions that appear once or twice next to the references. Whether this is a typo or a proper expression, these expressions are probably negligible.

Let RenkVec be a vector structured as two fields (str, float); RenkVec.str be PTRE string, and RenkVec.float be the rank of RenkVec.str.

Foreach *str ∈ SSV* do:

*RankVec*.*str ← SSV*.*str*
*RankVec*.*float ← CGER(x*,*y)*

done

### New (Internal) independent phrases scoring

We use CGER (sub-section 4.3.1), which considers the entire corpus, to make the first filtering and rating. Now we have a list of important PTREs with respect to the entire corpus, but we can filter them to a greater extent with respect to the "near-reference" expressions (i.e., PTREs), and rate them accordingly. The idea is to measure an expression according to its level of uniqueness. A unique phrase is an expression made up of a series of words, and none of the words appears in the entire list of the CGER expressions. The higher the level of its uniqueness, the higher its value will be. This means that this expression is important at the corpus level (CGER extraction), but it is also relatively important to the list of CGER expressions. This is important to note that in our case a single word is also considered a phrase, PTRE, (e.g. late) and we wanted to weight also a single word (how unique the word is relative to the rest of the PTRE). Therefore, we formulated the Independent Phrases Scoring (*IPS*) function. In the CGER phrases list, the IPS finds the dominant phrases to give them the highest scores (an explanation and an example are given below). The IPS scores a phrase as a log of the number of its appearances near references divided by its cornerstones, i.e., the words of the phrase, the IPS function ranges from -∞ to +∞.

Let *E* be the set of all phrases that remain after the filtering, i.e., *RenkVec*.*str*.

Let *e* be a phrase within *E*, i.e., *e ∈ E*.

Let *|e|* be the number of words within *e*.

Let *#e* be the number of times that e appears near reference.

Let *w ∈ e* be a word in phrase *e*.

Let *#w_e_* the number of phrases *e ∈ E* that the word *w* appears in.


$$IPS(e)={log}_{2}\frac{\#e_{j}}{\sum_{i}{\#w}_{e_{i}}}$$ (2)

For instance: The phrase *e = “blessed memory”* appears 170 times near references.

The word *“blessed”* appears in 7 different phrases *e* (including the phrase *“blessed memory”*).

The word *“memory”* appears in 13 different phrases *e* (including the phrase *“blessed memory”*).


$$IPS(e)={log}_{2}\frac{170}{7+13}=3.09$$

The denominator of the equation consists of the number of occurrences of each of the words that construct the expression; the larger the denominator, the smaller the result. The more important the phrase (high numerator) and the more unique the expression (low denominator), the higher the IPS score will be.

## Experimental results

The documents of the examined corpus were downloaded from the Bar-Ilan University’s Responsa Project (Contained in the Global Jewish Database (The Responsa Project at Bar-Ilan University). https://www.responsa.co.il/home.en-US.aspx.). The examined corpora include 15,450 responsa, which contain 24,930,082 words written by 24 scholars. This equates to an average of 643 files for each scholar. These authors lived over 229 years (1786–2015). These responsa contain many citations that were marked, as explained previously (section 3); each citation pattern can be expanded into many other specific citations [54].

First, we extract PTREs from the 15,450 responsa files (section 4.2) and receive 484,681 PTREs. Then, we ran the CGER function (section 4.3.1) on the 484,681 PTREs. The CGER function scored all the phrases and the filtering was performed by setting a threshold of 0.15%; as a result, the CGER function left us with 2,215 PTREs from 484,681 PTREs.

**Fig 2:**
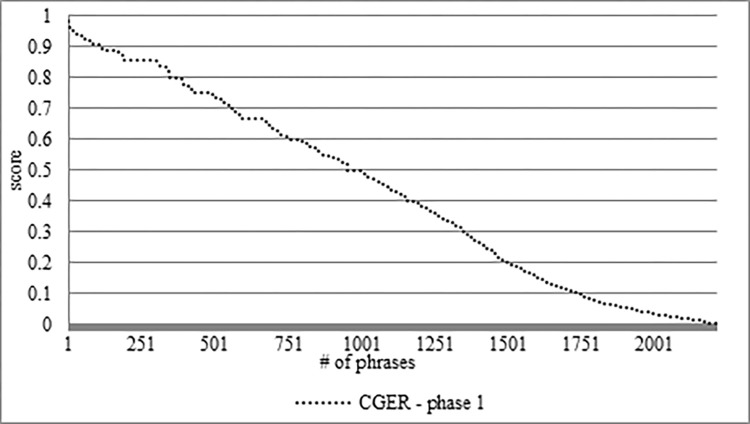
Ranking of 2,215 PTREs by CGER phase 1.

In Fig 2, we can see the CGER graph ranking of the 2,215 PTREs. In the range of the lowest 20%, there are 723 PTREs with a low ranking that represents approximately 32.6% of the PTREs. In the range of the highest 20%, there are 386 PTREs with a high ranking that represents approximately 17.4% of the PTREs.

Using CGER on the founded PTREs in the corpus gives us the first ranking for each PTRE. As previously mentioned, there may be sentences with references that we failed to mark, and the CGER treated them as sentences without references. To reduce the odds of this happening, after the first run of CGER (CGER phase 1), we decided to run it again with a slight change in the corpus. We took the highly rated PTREs (above 0.8) of the output of the CGER phase 1 as an indication for sentences that contain references that we missed/failed to markup. If a sentence with an unmarked reference contains two or more highly rated PTREs, we drop this sentence from the data set. The meaning of this is that if two PTREs with high rankings (by CGER phase 1) exist in an unmarked sentence, there is a high probability that this sentence has a reference that we failed to mark. After that, we run the CGER again (CGER phase 2) on a "cleaner" corpus and the results improved as shown later.

After running CGER phase 2 on the "cleaner" corpus, some of the PTREs gain a higher score.

**Fig 3:**
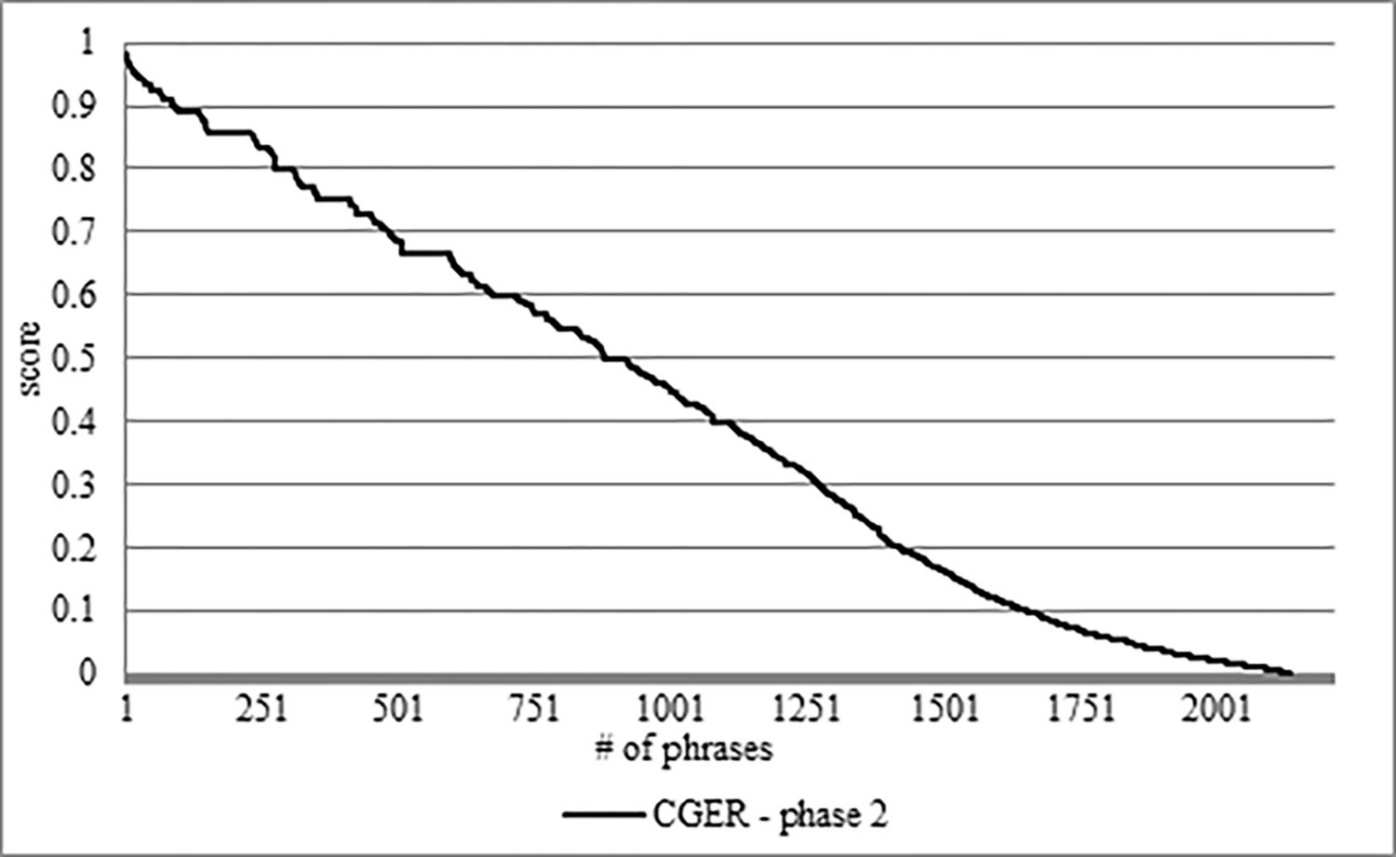
Ranking of 2,135 PTREs by CGER phase 2.

In Fig 3, we can see the rank of CGER phase 2 after filtering its output with a threshold of 0.15%. As a result, we left with 2,135 PTREs. CGER phase 2 indicates that it filtered 3.6% of the CGER phase 1 PTREs results. In the range of the lowest 20%, there are 716 PTREs with a low ranking that represents approximately 33.5% of the PTREs. In the range of the highest 20%, there are 308 PTREs with a high ranking that represents approximately 14.4% of the PTREs.

S2, S3 Appendixes present the TRE list. S2 Appendix shows only the TREs with a translation to English. S3 Appendix shows the grade changes between CGER phase 1 and CGER phase 2. S3 Appendix presents the 23 out of 44 TREs in CGER phase 2 that obtained a higher score than in CGER phase 1 (there is no decrease in the score of any of the TREs).

Since we could not find any study similar to ours (i.e., extraction of expressions of honor from which we find clues about time), we will refer to the CGER phase 2 as baseline (Note, we used as a baseline CGER phase 2 and not CGER phase 1 as a baseline; if we had treated CGER1 as a baseline the results would have been even better).

After running CGER phase 2, we used IPS (section 3.3.2) to narrow the search space even more and to rank the CGER phase 2 PTREs differently.

Following the CGER function that considers the entire corpus, we wanted to filter and rank the output of CGER phase 2 in a different manner. We wanted to only use the data that appears in the CGER phase 2 outputs, and for that, we run the IPS function. The IPS function score ranges from -∞ to +∞; a negative score means the phrase is not important, and a positive score means that the phrase is important. The higher the score, the more important the phrase is. We only took phrases with a positive score.

**Fig 4:**
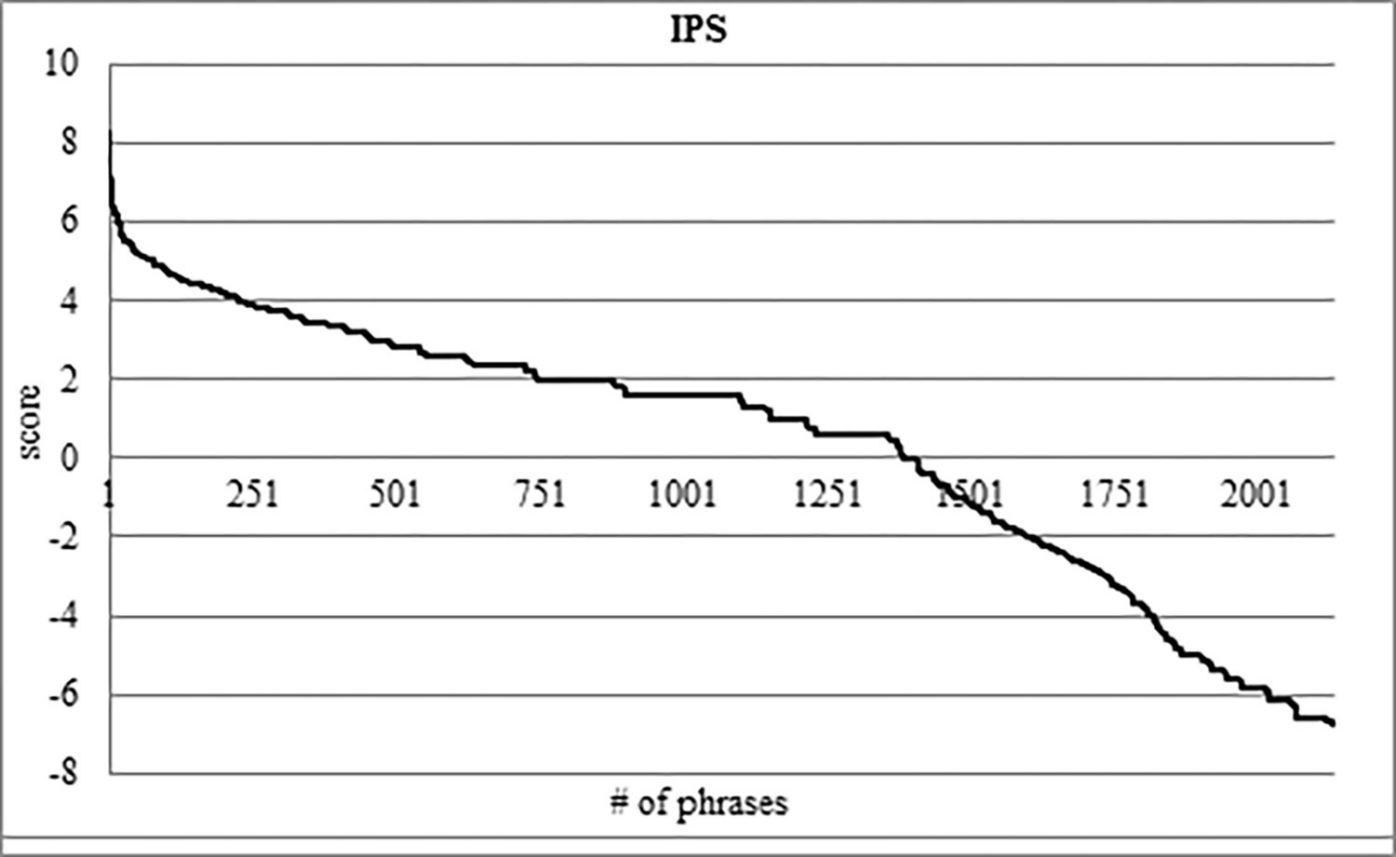
IPS score scale on the CGER phase 2 output.

In Fig 4, we can see the rank of IPS as a function of the CGER phase 2 filtering. IPS gives a positive score for 1,384 PTREs out of 2,135, i.e., IPS filtered 35.2% of expressions. Another important result is that almost all of the TREs receive a higher position in IPS than their location in CGER. This means that we not only narrowed the search space but also improved the location measure of the TREs. We will elaborate on this in section 5.2.

### Filtering the remaining PTREs

After the CGER filtering, we wanted to reduce the number of PTREs by a larger amount.

We looked for NLP tools but did not find such suitable tools for Hebrew. The quality of NLP tools designed for the Modern Hebrew language is notably inferior compared to the tools available for widely spoken Western languages such as English, Spanish, and German. This difference becomes notably accentuated when NLP is applied to rabbinic texts. These texts possess unique characteristics, encompassing sentences, expressions, and words originating from various eras (such as Bible, Mishnah, and Talmud). Each era exhibits its distinct style of expression and writing. Furthermore, rabbinic texts incorporate multiple languages, including Hebrew, Aramaic, and Yiddish. Presently, no NLP tools can handle this specific type of textual complexity; for this reason, we used grammar and heuristics for further filtering.

#### Grammatical screening

Because of the richness of the morphology of the Hebrew language, many words have grammatical affixes. In many cases, the affixes function as stop words, and as a result, these words are not TREs. To avoid over-sifting, we screen expressions that contain words whose grammatical affixes have, at least, two letters. Examples of words with two letters affixes: prefixes–**וב**פוסקים (**and in the** halachic authorities), **ול**דעת (**and to the** opinion of); suffixes—נוהג**ין** (traditional behave **of a community**, Aramaic suffix), העתק**תי** (**I** copied). Examples of words with one letter affix (which we did not screen): prefixes–**ו**דאי (sure), **ו**יתר (gave up); suffixes—מהרי"**ו** (Rabbi Ya’akov ben Yehuda Weil), ירושלמ**י** (Jerusalem Talmud). None of the "one function letters" are a function letter in the examples.

#### Grammatical Acronyms screening

Acronyms are a union of several words into one word. In Hebrew texts in general, and especially in rabbinical texts, acronyms are used very frequently. The initials in rabbinic texts have additions of grammatical prefixes that function as stop words. These acronyms cannot be time expressions because of the stop words, so we wipe out phrases that contain such acronyms. Examples of acronyms with prefixes: **דב**שו"ת (**which appears in the** questions and answers), **דל**פמ"ש (**according to** what was written).

#### Heuristic screening

The apostrophe ("`") role is to shorten a single word and leave it without a suffix, e.g., גב’ (Ms.), וכו’ (etc.). Many of the time expressions are composed of acronyms, or several words, which means that they appear in the text as a sequence of words. Because there are almost no (if any) TREs containing "`" it is reasonable to screen out phrases containing "`".

### Analyzing the results

We need to extract TREs intelligently. Our primary goal in these ranking methods is to intelligently narrow the search space while minimizing the “good” phrases that are being dropped out. In the outcome of CGER phase 1, we found 44 TREs. In the output of CGER phase 2, we found the same 44 TREs, which means that CGER phase 2 kept the TREs that were found by CGER phase 1. In S3 Appendix, we can see that CGER phase 2 did not only keep the TREs but also improved the ranking of 23 of 44 TREs (the rest remained unchanged). For example, there is a good improvement in the "זכרונו לברכה" (of blessed memory) expression; the rank climbed from 0.89 to 0.92, which is an improvement of 3.4%. However, the "זצוק"ל" (acronym: may the righteous and holy be of blessed memory) expression showed no change at all. Despite the improvement in the ranking of the 23 TREs, the internal order between the TREs remains the same. In other words, from the 44 TREs, the first phrase in CGER phase 1 was also the first phrase in CGER phase 2, as well as all the other TREs. All of this indicates the importance of the 44 TREs according to the CGER function.

The IPS scoring reduces the searching space of the PTREs found by CGER phase 2 by 35.7%, from 2,135 to 1373 phrases. In CGER phases 1 and 2, the number of TREs that were located in the upper half (according to CGER rank) was only 16 phrases i.e., 36.4%. In IPS scoring, there were 24 TRE phrases located in the upper half, i.e., 54.5%. We conclude not only that the IPS function reduces the search space for TREs, but that they also ranked the TREs much higher.

### Screening analysis

Now we will show the effects of grammatical and heuristic screening compared to the CGER phase 2 results.

We ran the CGER phase 2 function, which left us with 2,135 PTREs. Among these 2,135 PTREs, there are 44 TREs. We used the IPS function to filter the CGER 2,135 PTREs, and that left us with 1,373 PTREs; this demonstrates a 35.7% screening. Among these 1,373 PTREs, there are 41 TREs, which means that the IPS also screens three TREs. The three TREs screened by the IPS are 6.8% of the 44 TREs. The total number of the 44 TREs close to a reference is 9,543, and the total number of the three dropped TREs close to a reference is 12, i.e., 0.13%. This means that the importance of the three dropped TREs is negligible.

**Fig 5:**
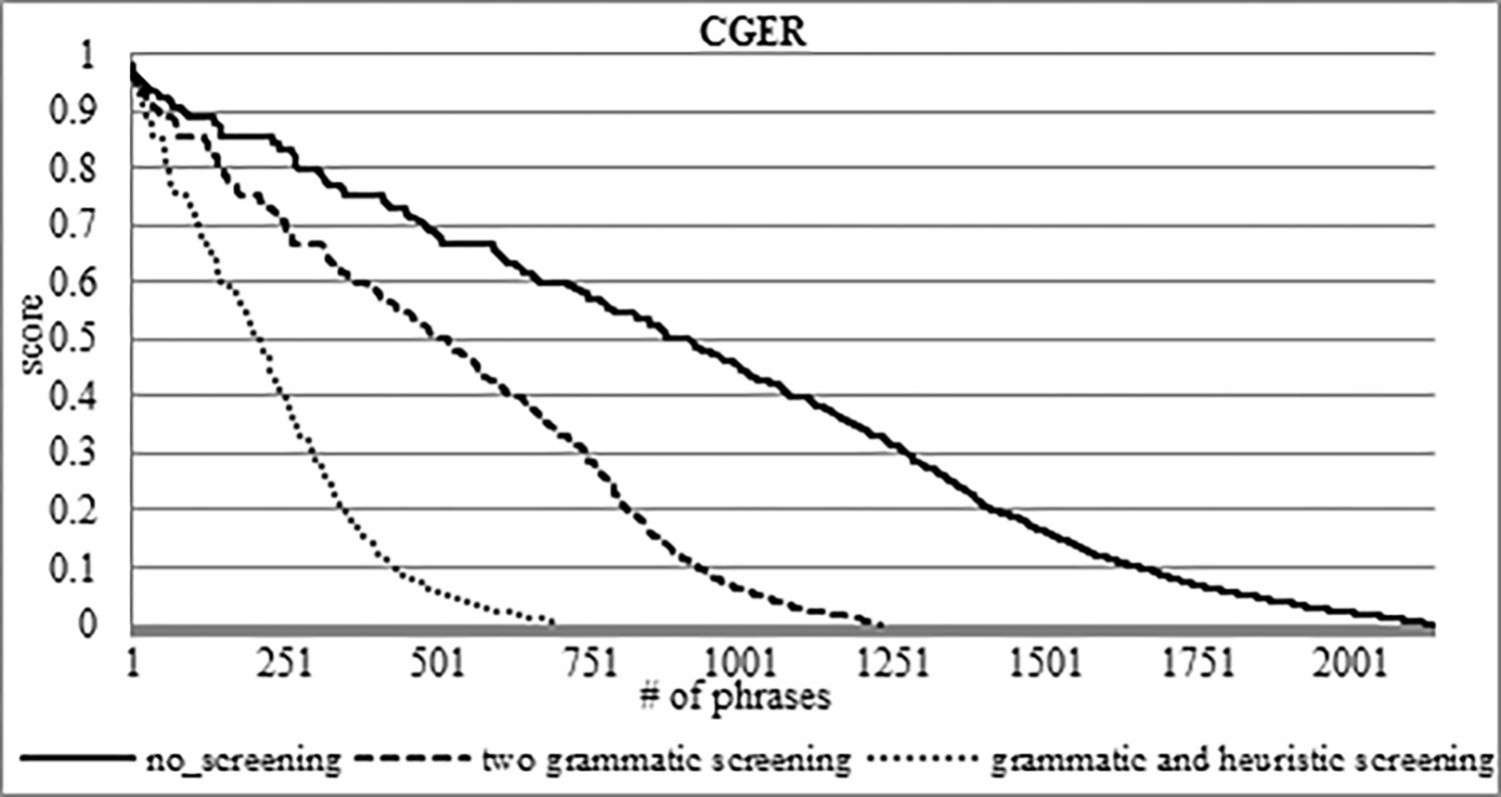
CGER scoring as a function of screening.

**Fig 6:**
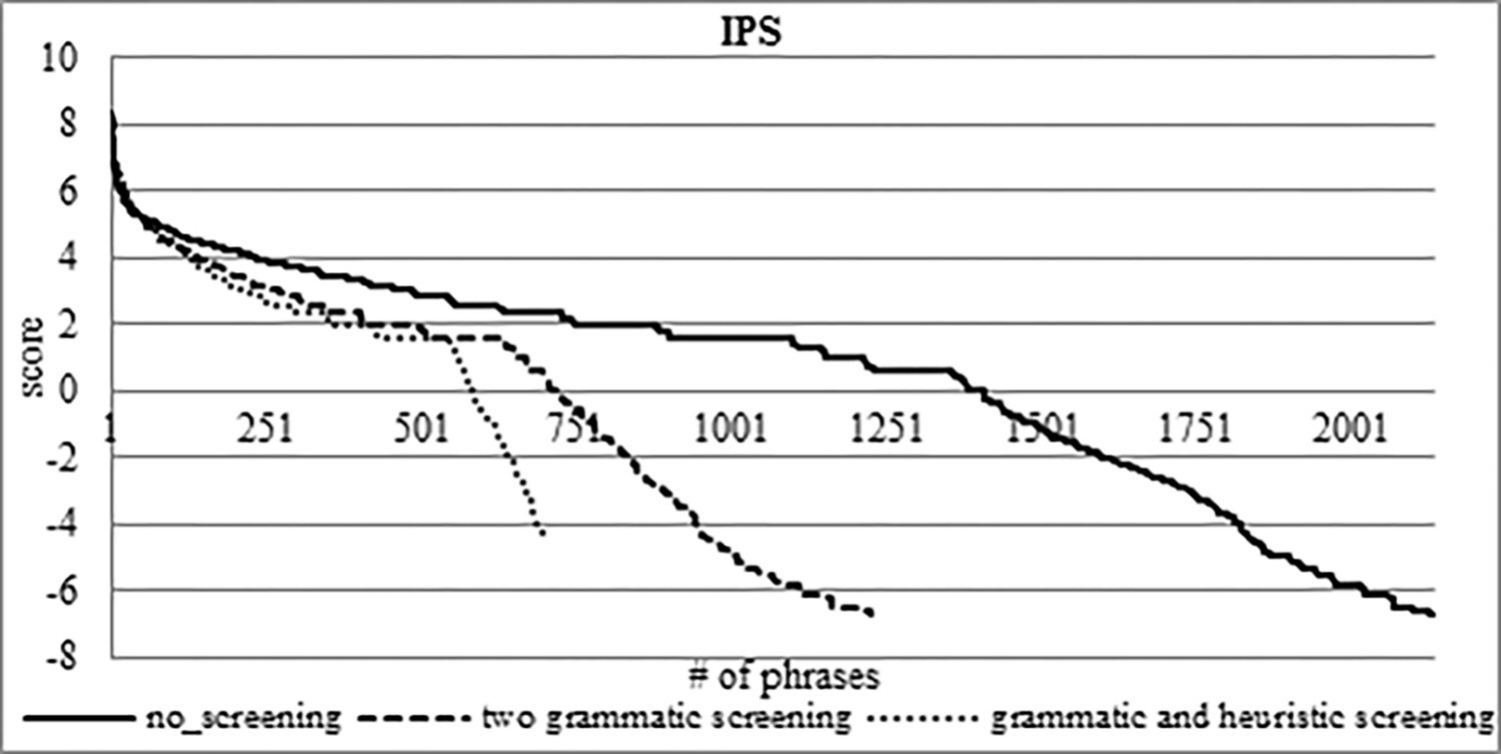
IPS scoring as a function of screening.

Next, we took the 2,135 PTREs (the CGER phase 2 output) and screened them with the two grammatical screenings, as previously mentioned, see Fig 5. After the two grammatical screenings, we ran the CGER function and obtained 1,224 PTREs, which is a decrease of 42.7% from the previous 2,135 PTREs. On those 1,224 PTREs, we ran the IPS function and obtained 708 PTREs, which is a decrease of 48.4% compared to the previous IPS screening, see Fig 6. In both functions (CGER and IPS), we lost six TREs from the 44 TREs, i.e., 13.6%, which means we are left with 38 TREs. The total count of the 44 TREs close to a reference is 9,543, and the total number of the dropped TREs (from 9,543) is 91, i.e., 0.95%. This means that the importance of the six dropped TREs is very low.

After we used grammatical properties for screening, we also applied heuristic screening (section 5.1). We took the 1,224 PTREs (the result of the grammatical screening) and applied apostrophe ("`") heuristic screening. With the CGER function, we are left with 697 PTREs, a decrease of 43.1% from the previous 1,224 PTREs, see Fig 5. On those 697 PTREs, we the IPS function and it left us with 575 PTREs which means a decrease of 18.8% compared to the previous IPS screening, see Fig 6. One of the important things is that heuristic screening is that we did not drop-out any of the 38 TREs that we had, which proves the quality of the heuristic, Figs 5 and 6 show the progress of the screening process.

Summary: running the CGER function filtered 99.6% of the PTREs, which left us with 2,135 PTREs and 44 TREs. Then, after we ran the IPS function and grammatical and heuristic screening, we lost six unimportant TREs and were left with only 575 PTREs, i.e., 0.12% compared to the beginning.

**Fig 7:**
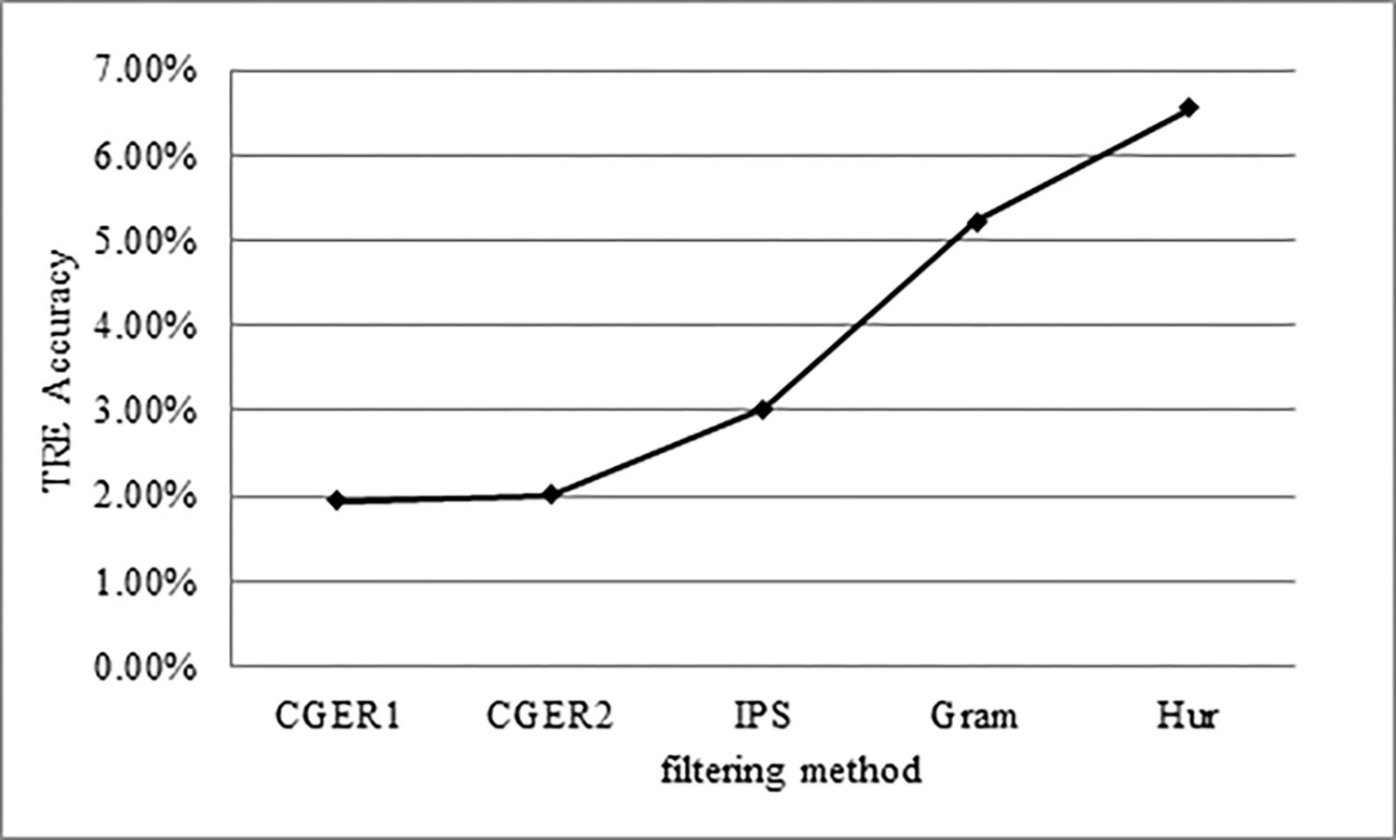
TRE accuracy over the filtering process.

We have developed methods for extracting TREs. Because TREs appear next to citations we extracted phrases/words that appear next to citations. In order to filter and retrieve only expressions that are next to citations that do not appear in the rest of the text (phrases/words unique to the citation environment), we have developed two statistical functions. The first refers to the proportion between the presence of expressions that are next to citations and their presence in the rest of the text. Phrases/words that appear almost exclusively next to citations will get a high score and those that are almost exclusively in the rest of the text will get a low score. We went through the phrases/words with high weight and we found the TREs. In order to further narrow down the search space (which we need to go through manually) we performed three more steps, (1) activating the IPS on the results of the CGER, (2) activating linguistic filtering, and (3) activating heuristics filtering. Indeed, the results are encouraging:

CGER’s initial filtering decreased the number of phrases/words from 484,681 phrases/words to 2215 phrases/words, of which the accuracy is 1.94% TRE. Fig 7 shows the increase in accuracy of the TREs as a result of the filtering process.

1. The IPS decreased the number of phrases/words from 2215 to 1373 phrases/words, of which the accuracy is 3.03% TRE.
2. The Linguistic filtering decreased the number of phrases/words from 1373 to 708 phrases/words, of which the accuracy is 5.22% TRE.
3. Finally, the heuristic filtering decreased the number of phrases/words from 708 to 575 phrases/words, of which the accuracy was 6.55% TRE.

To sum up: we see that as we do more filtering we lower the manual search space and also get higher accuracy, see Fig 7.

**Fig 8:**
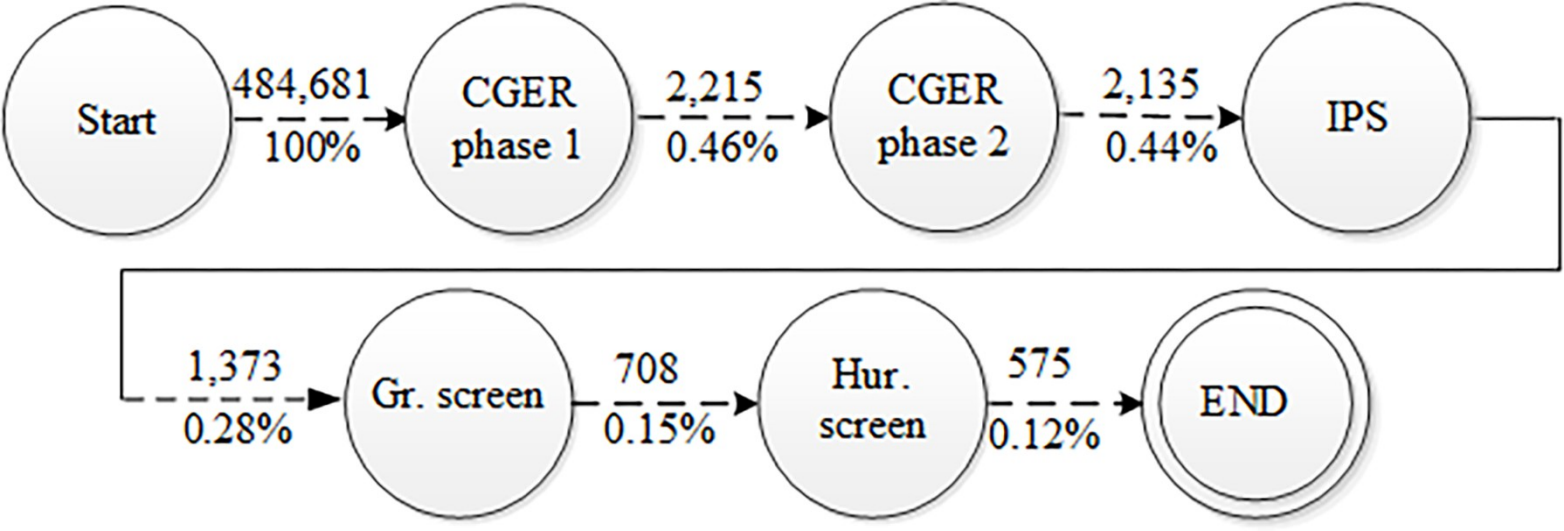
Results over the entire process.

Fig 8 shows the overall filtering process and the amount of PTREs that were left from the TRE search space.

When we ran the algorithm (section 4.2), we obtained 484,681 phrases. On those 484,681 phrases, we applied the CGER formula (CGER phase 1) for the first time. The CGER formula filtered 95.6% of the phrases, which left us with 2,215 phrases. On the output of CGER phase 1, we applied the CGER formula (CGER phase 2) for the second time, which filtered more than 80 phrases. On the output of CGER phase 2, we ran the IPS formula and decreased the PTREs by 35.7% to 1,373 phrases. After using the statistical formulas, we moved to use the grammatical and heuristical screening (on the results of the IPS formula). We used the phrases that the IPS formula left and screened them with grammatical properties (section 5.1). The grammatical screening dropout was 48.4% of the phrases, and we were left with 708 PTREs. In the end, we took those 708 PTREs and screened them with an apostrophe ("`") heuristic (section 5.1), and were left with only 575 phrases.

Interim summary: Compared to the baseline (i.e., CGER phase 2) IPS filtered 762 of these expressions, leaving 1,373. This represents a 35.7% reduction in the number of expressions. IPS also removed two TREs, a 4.5% (2/44) decrease.

In the Linguistic filtering stage, we removed 665 expressions, a 48.4% (665/1,373) reduction. This also resulted in the removal of six TRE, a 14.3% (6/42) decrease.

Finally, in the heuristic filtering stage, we removed 133 expressions, an 18.8% (133/708) decrease. No TRE where removed in this step.

Overall, we were able to filter 73.1% (1,560) of the PTRE from the baseline. Among these removals, on the one hand, we lost eight TREs, which is 18.2% of the 44 TREs, on the other hand, it is only 0.5% of the 1,560 removed PTRE.

We calculate the precision, recall, and F1-score with the assumption that the total of 44 TREs (which is what appeared in the CGER phase 1 results).

1. CGER’s initial filtering decreased the number of phrases/words from 484,681 phrases/words to 2215 phrases/words, of which the accuracy of TRE is 1.99%, precision: 0.0206, recall: 1 → F1 = 0.0389
2. CGER phase 2 decreased the number of phrases/words from 2215 to 2135 phrases/words, of which the accuracy of TRE is 2.06%, precision: 0.0206, recall: 1→ F1 = 0.0404
3. The IPS decreased the number of phrases/words from 2135 to 1373 phrases/words, of which the accuracy of TRE is 3.06%, precision: 0.0306, recall: 0.9545 → F1 = 0.0593
4. The Linguistic filtering decreased the number of phrases/words from 1373 to 708 phrases/words, of which the accuracy of TRE is 5.08, precision: 0.0508, recall: 0.8182 → F1 = 0.0957
5. Finally, the heuristic filtering decreased the number of phrases/words from 708 to 575 phrases/words, of which the accuracy of TRE is 6.26, precision: 0.0625, recall: 0.8182 → F1 = 0.1161

**Fig 9:**
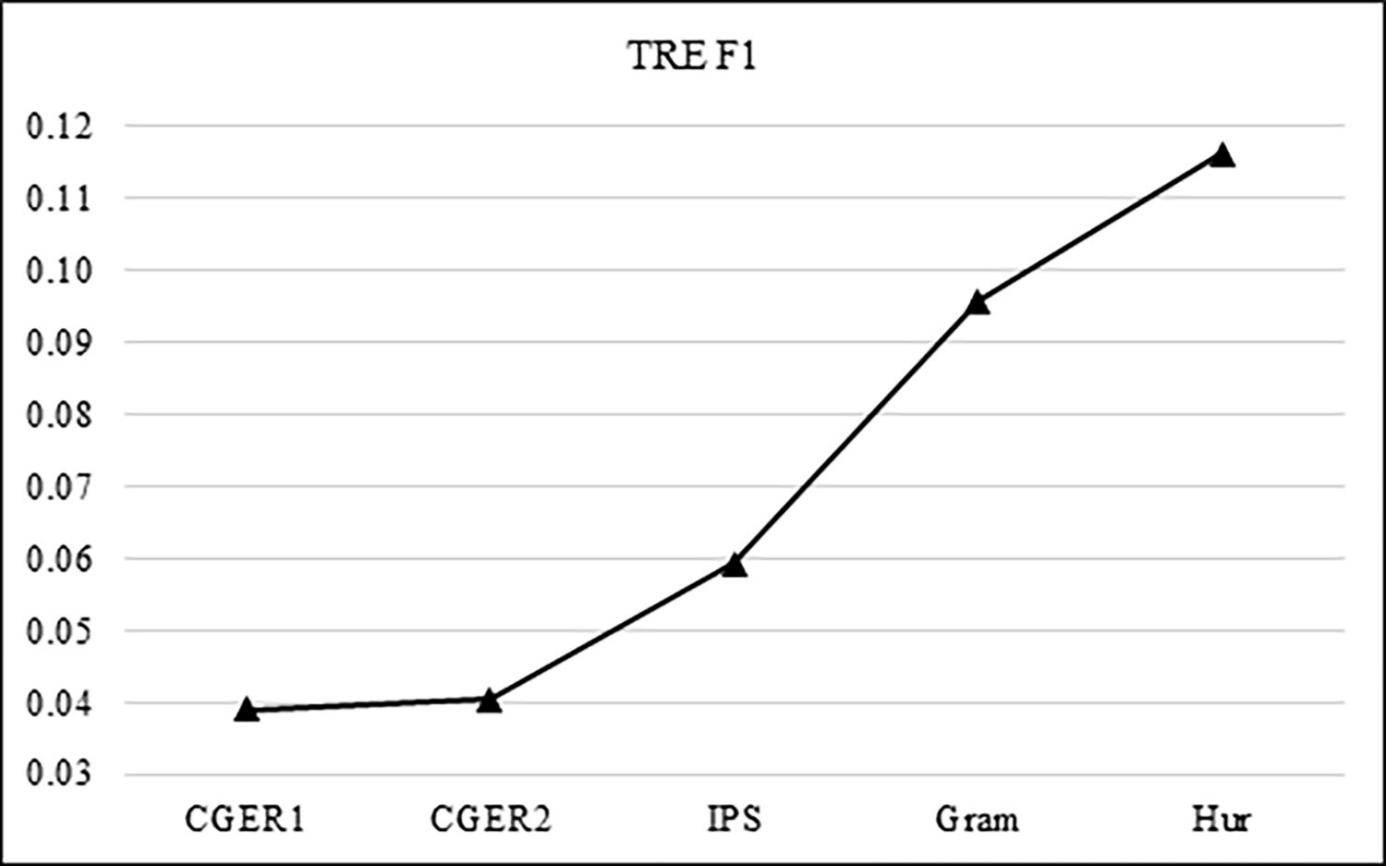
TRE F1 over the filtering process.

Fig 9 shows the progress of the F1 results of the TRE as a function of the filtering process.

From another point of view, the role of the filtering process is to delete phrases that are not relevant, when we quantify the success of the filtering process then: precision is 1,552/1,560 = 0.995, recall is 1,552/2,091 = 0.742 → F1 = 0.85

In summary, we started with 484,681 phrases and ended up with only 575 phrases. We are left with only the important phrases, which are 0.12% of the initial phrases. The most influential step was the CGER phase 1 function, which in one step dropped 99.54% of the expressions. The following steps continue to filter and rank the PTREs. Although the number of TREs is not large, the process we did was able to filter out 99.88% from the 484,681 PTREs that we began with.

### Error analysis

Most of the errors in the output occur because of (1) our failure to mark some references or (2) marking part of a reference but not all of it, i.e., not because of the failure of the filtering functions. One of the phenomena of response texts is the appearance of references in a sequence next to each other. Because we failed to mark some of them, marked reference(s) appear next to reference(s) that we failed to mark, then the function flags the unmarked reference as a “next to reference” phrase (see examples below). We will elaborate on the two types of the most common mistakes.

(1) Examples for the first error: the bold are marked references and the italicized are unmarked references that the function outputs as a "next to reference" phrase.

שו"ת אחיעזר חלק ג סימן ל

… **והרי"ף והרמב"ם והרא"ש** *והטוש"ע* השמיטו התוספתא, וגם בדברי…

### Responsa Ahiezer Part 3 chapter 30

… and the **Ri”f** (acronym: rabbi Yitzchak Alfasi) and the **Ramba"m** (acronym: Rabbi Moshe ben Maimon) and the **Ros”h** (acronym: Our rabbi Asher) and the *tosh”a`a* (acronym: Tur Shulchan Aruch) discard the Tosefta i.e., supplement to the Mishnah, and also in the writing of …

(2) Examples for the second error: the bold are marked references and the italicized are parts of the unmarked references. In this part, we can see the length and the complexity of the references in this kind of text.

שו"ת ב"ח החדשות סימן מז

… לא משמע הכי בדברי **הרמב"ם** *פ"א מהל’ ת"ת* דאע"פ שכת’ כשם שחייב אדם ללמוד את בנו…

### Responsa the New B”h (Bait Hadash) chapter 47

… this is not the meaning of the words of the **Ramba"m** (acronym: Rabbi Moshe ben Maimon) *C”A of Hil`T”T* (acronym and abbreviation: chapter A of the halachot (religious laws) Talmud Torah) even though he wrote as a man must teach his son …

A review of the removed TREs shows that these expressions are not distinct expressions of respect and time, for example:

הראשונים (harishonim)—in the context we are looking for it is "medieval biblical commentators", however, the meaning of this phrase is founders, pioneers, the first ones, the formers, etc`, therefore, this phrase is less relevant to our issues.

ואביו (and his father)—an expression used, among other things, to describe entities in halachic cases, is less an expression of respect and less indicative of time in our context.

נאם הצעיר (Neum the young)—When some rabbis finish a halachic answer or a halachic discussion they use the phrase “Neum the young” as part of their signature.

## Summary, conclusions, and future work

We have shown an unsupervised process that extracts TREs from rabbinic texts in a semi-automatic manner. To achieve that goal, we decided to look for them in the most likely place they should appear in a rabbinic text next to rabbinic names / nicknames / acronyms / book-names.

The extraction process started with PTRE extraction from around rabbinic references. Then, we used those PTREs and counted the number of their appearances near rabbinic citations in the entire corpus. We ran the CGER function in two phases to filter and rank the extracted PTREs. We ran the IPS function on top of the CGER function results. We added two screening stages (grammatical and heuristic) and the results were very good in the end; we were able to filter out and rank 575 phrases that were the final PTREs from approximately half a million phrases. From this set, we extracted 38 real TREs, of which most achieved a high score at the end.

Our contributions include the following: extracting time-related keyphrases (which, in our case, are actually expressions of honor from which we find clues about time) from a small text window near citations, developing two new statistical formulas that grade and filter expressions (the good results we have obtained prove the quality of these functions), we are the first to extract time-related keyphrases in an unsupervised method from highly morphological Semitic languages, and we use the complex morphology of the Semitic languages to our advantage, with a unique use of the grammatical affixes as a tool to filter data. The extraction of time-related phrases is important. For example, it can be used to date texts and, in some cases, can help to identify unknown writers.

We also plan to apply deep learning methods to construct a vector space model to create fixed-size vectors that represent expressions that do not necessarily have the same number of words. From these vectors, we will construct clusters of expressions, some of which will be clusters of TREs.

## Supporting information

S1 Table # of PTRE and TRE removed over the process compared to the baseline. (DOCX)

S1 Appendix Details about the data set and its authors. (DOCX)

S2 Appendix Translation of the 44 TRE list. (DOCX)

S3 Appendix Ranking improvements of 44 TREs from CGER phase 1 to CGER phase 2. (DOCX)
