# Supplementary material for: Extraction of time-related expressions using text mining with application to Hebrew
Source: PLoS One. 2024 Feb 23;19(2):e0293196. doi: 10.1371/journal.pone.0293196 (PMC10889890; doi:10.1371/journal.pone.0293196)
Supplement: S1 Table — (DOCX) [file pone.0293196.s001.docx]

Table S1. # of PTRE and TRE removed over the process compared to the baseline

| **function** | **# PTRE Exists** | **# PTRE Filtered** | **# TRE Removed** | **% PTRE Filtered** | **% TRE Removed** |
| --- | --- | --- | --- | --- | --- |
| **Baseline** | 2,315 | NA | NA | NA | NA |
| **IPS** | 2,315 | 762 | 2 | 35.7% | 4.5% |
| **Linguistic Filtering** | 1373 | 665 | 6 | 48.4% | 14.3% |
| **Heuristic Filtering** | 708 | 133 | 0 | 18.8% | 0% |
| **Overall** | 575 | 1560 | 8 | 73.1% | 18.2% |
