# Supplementary material for: Extraction of time-related expressions using text mining with application to Hebrew
Source: PLoS One. 2024 Feb 23;19(2):e0293196. doi: 10.1371/journal.pone.0293196 (PMC10889890; doi:10.1371/journal.pone.0293196)
Supplement: S1 Appendix — (DOCX) [file pone.0293196.s002.docx]

**Appendix A**

|  | **Details about the data set and its authors** | | | | | |
| --- | --- | --- | --- | --- | --- | --- |
| **#** | **Author's name** | **Birth year** | **Death year** | **# of files** | **# of words** | **# of chars** |
| 1 | Vozner Shmuel | 1914 | 2015 | 1807 | 1,490,463 | 7,768,059 |
| 2 | Yosef Ovadya | 1920 | 2014 | 1283 | 4,578,049 | 22,933,473 |
| 3 | Waldenberg Eliezer | 1917 | 2006 | 1639 | 3,197,662 | 16,589,888 |
| 4 | Auerbach Shlomo Zalman | 1910 | 1995 | 229 | 793,706 | 4,087,592 |
| 5 | Weiss Yitzchak | 1902 | 1989 | 1468 | 2,311,927 | 11,695,021 |
| 6 | Stern Bezalel | 1911 | 1989 | 663 | 1,080,452 | 5,390,661 |
| 7 | Feinstein Moshe | 1895 | 1986 | 1831 | 2,306,526 | 11,959,224 |
| 8 | Hadaya Ovadia | 1890 | 1969 | 210 | 713,341 | 3,683,787 |
| 9 | Ades Yaakov | 1898 | 1963 | 131 | 310,585 | 1,604,218 |
| 10 | Havita Rahamim | 1901 | 1959 | 736 | 898,543 | 4,655,681 |
| 11 | Herzog Yitzchak | 1889 | 1959 | 190 | 430,259 | 2,210,586 |
| 12 | Ben-Zion Meir Hai Uziel | 1880 | 1953 | 374 | 899,617 | 4,621,414 |
| 13 | Boimel Yehoshua | 1880 | 1948 | 129 | 237,093 | 1,227,007 |
| 14 | Baer Weiss Yitzchak | 1873 | 1942 | 497 | 243,789 | 1,257,633 |
| 15 | Kook Abraham Yitzchak | 1865 | 1935 | 681 | 750,145 | 3,892,610 |
| 16 | Allouch Faraji | 1854 | 1921 | 112 | 205,258 | 1,069,460 |
| 17 | Schwadron Sholom Mordechai | 1835 | 1911 | 1574 | 1,657,860 | 8,560,084 |
| 18 | Somekh Abdallah | 1813 | 1889 | 86 | 80,508 | 412,486 |
| 19 | Spektor Yitzchak Elchanan | 1817 | 1896 | 301 | 1,159,019 | 5,843,696 |
| 20 | Trunk Israel Yehoshua | 1820 | 1893 | 281 | 132,257 | 689,598 |
| 21 | Abuhatzeira Yaakov | 1790 | 1880 | 146 | 177,411 | 917,682 |
| 22 | Edery Abraham | 1801 | 1874 | 119 | 176,849 | 918,564 |
| 23 | Assad Yehuda | 1794 | 1866 | 882 | 880,361 | 4,565,230 |
| 24 | Birdugo Yaakov | 1786 | 1843 | 126 | 218,402 | 1,130,206 |
