# Supplementary material for: Extraction of time-related expressions using text mining with application to Hebrew
Source: PLoS One. 2024 Feb 23;19(2):e0293196. doi: 10.1371/journal.pone.0293196 (PMC10889890; doi:10.1371/journal.pone.0293196)
Supplement: S2 Appendix — (DOCX) [file pone.0293196.s003.docx]

Appendix B

| **Translation of the 44 TRE list** | |  |  |
| --- | --- | --- | --- |
| **Translation of the TREs** | **TREs in Hebrew** | **#** |  |
| Love him (acronym:) with heart and soul | אוהבו בלו"נ | 1 | refers to living humans |
| Love you (acronym:) with heart and soul | אוהבך בלו"נ | 2 |  |
| acronym: may G-D preserve him | ה"י | 3 |  |
| The genius | הגאון | 4 |  |
| The genius Rabbi | הגאון רבי | 5 |  |
| acronym: may G-D preserve him amen | הי"א | 6 |  |
| acronym: may G-D preserve him and grant him life | הי"ו | 7 |  |
| the Young | הצעיר | 8 |  |
| acronym: The Rabbi the genius | הרה"ג | 9 |  |
| acronym: The Rabbi the genius Rabbi | הרה"ג רבי | 10 |  |
| acronym: and his candle will shine | והנ"י | 11 |  |
| his dear friend | ידידו מוקירו | 12 |  |
| acronym: may G-D preserve him and grant him life | יצ"ו | 13 |  |
| acronym: the Honorable our teacher the Rabbi | כמה"ר | 14 |  |
| acronym: the Honorable our teacher the Rabbi | כמוה"ר | 15 |  |
| acronym: Our teacher the Rabbi | מהר"ר | 16 |  |
| acronym: My Teacher and my Rabbi | מו"ר | 17 |  |
| acronym: My Teacher and my Rabbi may he live a good long life, Amen | מו"ר שליט"א | 18 |  |
| acronym: his candle will shine | נ"י | 19 |  |
| acronym: soul spirit | נ"ר | 20 |  |
| Neum the young | נאם הצעיר | 21 |  |
| acronym: may G-D preserve him and grant him life | נר"ו | 22 |  |
| acronym: His end will be good. Amen | סילט"א | 23 |  |
| The splendor of the generation | פאר הדור | 24 |  |
| My Rabbi | רבי | 25 |  |
| acronym: may he live a good long life, Amen | שליט"א | 26 |  |
| The first ones (acronym:) of blessed memory | הראשונים ז"ל | 27 | Refers to deceased persons |
| acronym: of blessed memory | ז"ל | 28 |  |
| acronym: May his virtue stand for us | זי"ע | 29 |  |
| acronym: May his virtue stand for us amקn | זיע"א | 30 |  |
| of blessed memory | זכרו לברכה | 31 |  |
| of blessed memory | זכרונו לברכה | 32 |  |
| acronym: His memory of the World to Come | זלה"ה | 33 |  |
| acronym: of his blessed memory for the life of the World to Come | זללה"ה | 34 |  |
| acronym: may the righteous be of blessed memory | זצ"ל | 35 |  |
| acronym: may the righteous and holy be of blessed memory | זצוק"ל | 36 |  |
| acronym: may rest in peace | ע"ה | 37 |  |
| My father | אבי | 38 | Known whether they are deceased or alive |
| My son | בני | 39 |  |
| The first ones | הראשונים | 40 |  |
| and his father | ואביו | 41 |  |
| and my master my father | ואדוני אבי | 42 |  |
| my grandfather/elder | זקני | 43 |  |
| My student | תלמידי | 44 |  |
