# Supplementary material for: Extraction of time-related expressions using text mining with application to Hebrew
Source: PLoS One. 2024 Feb 23;19(2):e0293196. doi: 10.1371/journal.pone.0293196 (PMC10889890; doi:10.1371/journal.pone.0293196)
Supplement: S3 Appendix — (DOCX) [file pone.0293196.s004.docx]

Appendix C

Ranking improvements of 44 TREs from CGER phase 1 to CGER phase 2
